# Osthole ameliorates hepatic fibrosis and inhibits hepatic stellate cell activation

**DOI:** 10.1186/s12929-015-0168-5

**Published:** 2015-08-01

**Authors:** Ya-Wei Liu, Yung-Tsung Chiu, Shu-Ling Fu, Yi-Tsau Huang

**Affiliations:** Institute of Traditional Medicine, School of Medicine, National Yang-Ming University, No. 155, Li-Nong Street, Sec. 2, Taipei, 11221 Taiwan; Department of Medical Research and Education, Taichung Veterans General Hospital, No. 1650, Taiwan Boulevard Sec. 4, Taichung, 40705 Taiwan; National Research Institute of Chinese Medicine, Ministry of Health and Welfare, No. 155-1, Li-Nong Street, Sec. 2, Taipei, 11221 Taiwan

**Keywords:** *Cnidium monnieri* (L.) Cusson, Osthole, Hepatic fibrosis, Hepatic stellate cells, Inflammation

## Abstract

**Background:**

Hepatic fibrosis is a dynamic process which ultimately leads to cirrhosis in almost patients with chronic hepatic injury. However, progressive fibrosis is a reversible scarring response. Activation of hepatic stellate cells (HSCs) is the prevailing process during hepatic fibrosis. Osthole is an active component majorly contained in the fruit of *Cnidium monnieri* (L.) Cusson. This present study investigated the therapeutic effects of osthole on rat liver fibrosis and HSC activation.

**Results:**

We established the thioacetamide (TAA)-model of Sprague–Dawley (SD) rats to induce hepatic fibrosis. Rats were divided into three groups: control, TAA, and TAA + osthole (10 mg/kg). *In vivo*, osthole significantly reduced liver injury by diminishing levels of plasma AST and ALT, improving histological architecture, decreasing collagen and α-SMA accumulation, and improving hepatic fibrosis scores. Additionally, osthole reduced the expression of fibrosis-related genes significantly*.* Osthole also suppressed the production of fibrosis-related cytokines and chemokines. Moreover, nuclear translocation of p65 was significantly suppressed in osthole-treated liver. Osthole also ameliorated TAA-induced injury through reducing cellular oxidation. Osthole showed inhibitory effects in inflammation-related genes and chemokines production as well. *In vitro*, we assessed osthole effects in activated HSCs (HSC-T6 and LX-2). Osthole attenuated TGF-β1-induced migration and invasion in HSCs. Furthermore, osthole decreased TNF-α-triggered NF-κB activities significantly. Besides, osthole alleviated TGF-β1- or ET-1-induced HSCs contractility.

**Conclusions:**

Our study demonstrated that osthole improved TAA-caused liver injury, fibrogenesis and inflammation in rats. In addition, osthole suppressed HSCs activation *in vitro* significantly.

**Electronic supplementary material:**

The online version of this article (doi:10.1186/s12929-015-0168-5) contains supplementary material, which is available to authorized users.

## Background

Hepatic fibrosis is a dynamic wound-healing response to chronic hepatic injuries such as alcoholism and viral hepatitis in patients. Accumulated evidence suggests that hepatic fibrosis is a reversible process [1]. Activation of hepatic stellate cells (HSCs) is the prevailing process during hepatic fibrosis [2]. Activated HSCs generate fibrosis by accumulating extracellular matrix (ECM), secreting cytokines and chemokines, and enhancing the ability of chemotaxis [3]. Refraining quiescent HSCs from transformation is a crucial approach to impeding the fibrotic response in the liver.

Activated HSCs undergo phenotypic transformation with diverse functional changes, including fibrogenesis, chemotaxis, contractility, proliferation, cytokine secretion, and ECM degradation [4]. HSCs could be triggered to transdifferentiate from quiescent into activated form by inflammatory mediators or growth factors, such as tumor necrosis factor-α (TNF-α), tumor growth factor-β1 (TGF-β1) and endothelin-1 (ET-1) [5–7].

Osthole (C_15_H_16_O_3_, MW = 244.29) is an active component present in many medicinal plants especially in the fruit of *Cnidium monnieri* (L.) Cusson, which has long been used in clinics with pharmacological properties, such as anti-oxidation and anti-inflammation [8]. Many reports indicated that osthole improves hepatic steatosis, including reducing triglyceride synthesis [9, 10], hepatic inflammation [11] and modulating lipogenic gene expressions in the liver [12]. Evidence has shown that osthole also suppresses the *in vitro* secretion of hepatitis B surface antigen in a hepatoma cell line [13]. However, its anti-fibrotic effect in the liver still requires further investigation.

Thioacetamide (TAA, CH_3_CSNH_2_) administration is a well-accepted method to establish liver fibrosis in rats [14, 15]. TAA leads to hepatotoxicity by oxidation processes. The metabolites from TAA are more toxic than TAA and can result in the liver injury. Studies indicated that the pathology of TAA-induced liver injury is similar in some ways to that of cirrhosis in human [16].

In the present study, we investigated both the *in vivo* and *in vitro* effects of osthole in hepatic fibrotic rats induced by TAA, and HSCs activated by TNF-α, TGF-β1, or ET-1.

## Methods

### Drug preparation and chemicals

For *in vivo*, osthole (Selleck Chemicals, Boston, MA, USA, Additional file 1: Figure S1) was mixed with 0.7 % carboxyl methyl cellulose (CMC, Sigma-Aldrich, St. Louis, MO, USA) for administration. For *in vitro*, osthole was dissolved in DMSO and diluted with medium to give a DMSO concentration below 0.1 %. TGF-β1 (1 ng/ml), TNF-α (10 ng/ml) and ET-1 (1 nM, Sigma-Aldrich) were used for stimulation.

### Animal and experimental model of fibrosis

A total of 28 male SD-strain rats (8-week-old, National Yang-Ming University Experimental Animal Center) were housed with free access to food and water in an air-conditioned room at 21 °C with 12 h light–dark cycle. Hepatic fibrosis was induced by TAA (Sigma-Aldrich) dissolved in normal saline and injected intraperitoneally (i.p.) into rats twice a week at a dosage of 250 mg/kg for 6 weeks. At the beginning of week three, rats were randomly divided into three groups: (a) Control group: rats given saline injections and gavage of 0.7 % CMC (*n* = 8); (b) TAA group: rats given TAA injections and gavage of 0.7 % CMC (*n* = 10), (c) TAA + osthole group: osthole (10 mg/kg) was given by gavage twice daily for 4 weeks (*n* =10). Each rat was anesthetized by pentobarbital (50 mg/kg i.p.), then the whole liver was removed for measurements. Left-lobe sections were used for immunohistochemical studies by fixation (10 % formalin) and subsequent paraffin-embedment. Other liver tissues were used for isolating proteins and RNA. All animal studies were approved by the Animal Experiment Committee of the University (IACUC No: 98-111) and conducted humanely, in accordance with the Guide for the Care and Use of Laboratory Animals (National Academic Press, USA, 1996).

### Measurement of hepatic injury

Rat blood samples were obtained by heart puncture after anaesthesia. Plasma was separated and utilized for measuring alanine transaminase (ALT) and aspartate transaminase (AST) activities as markers of liver injury using a colorimetric analyzer (Dri-Chem 3000; Fuji Photo Film Co., Tokyo, Japan), as described previously [17].

### Histological examination and immunohistochemistry

Paraffin-embedded liver sections were deparaffinized and dehydrated in a graded alcohol series. Sections were stained with GM's Hematoxylin-eosin (H&E) dye (Muto Pure Chemicals Co. Ltd, Tokyo, Japan) or Picrosirius red kit (Polysciences, Inc., Warrington, PA, US) to evaluate inflammatory degree and collagen distribution, respectively. Immunohistochemical staining to detect α-SMA was performed by using the DAKO EnVision System (DAKO North America, Carpinteria, CA, USA) according to the manufacturer’s protocol. The degree of hepatic fibrosis was evaluated using Ishak fibrosis scoreby a pathologist (YC Chiu) in a blind fashion [18].

### Quantitative quantitative reverse transcription-polymerase chain reaction (qRT-PCR) analysis

Hepatic RNA was extracted by TRIzol® Reagent (Life Technologies, Carlsbad, CA, USA) according to the manufacture protocol, as previously reported by us [19]. RNA was reverse-transcribed into cDNA using oligo-dT and dNTP. PCR was performed by the StepOnePlus™ Quantitative Real-Time PCR System (Life Technologies), according to the manufacturer’s instructions. The sequences of primers for PCR are listed as supporting information (Additional file 2: Table S1).

### ELISA for cytokines

Rat plasma was used to determine the levels of cytokines and chemokines, including tissue inhibitor of metallopeptidase-1 (TIMP-1), intercellular adhesion molecule-1 (ICAM-1), CXCL7, CD62L, VEGF, CX3CL1, LIX, CXCL1, CCL20, and CCL5, using an Rat Cytokine Array Kit (R&D Systems, Minneapolis, MN, USA), according to the manufacturer’s specifications.

### Western blotting analysis

Liver protein extracts were harvested with RIPA buffer. Nuclear extracts were prepared according to our previous study [19]. Cell lysates were separated with SDS-PAGE and transferred onto PVDF. The following antibodies have been used in various dilutions: anti-α-SMA (Calbiochem-Novabiochem, San Diego, CA, USA), and anti-α-tubulin, anti-Nrf-2, anti-p65, anti-JunD (Santa Cruz Biochnology, Santa Cruz, California, CA, USA), and anti-PCNA (Cell Signaling Technology, Danvers, MA, USA). The HRP-conjugated secondary antibodies were used and blots were developed using ECL detection reagent and detection imaging system.

### Glutathione (GSH), 4-hydroxynonenal (4-HNE), and malondialdehyde (MDA) in liver tissue

Liver tissue samples (0.05 g) were washed with ice-cold 1X PBS containing heparin (0.16 mg/ml). Oxidized glutathione (GSSG) and total GSH were measured using Glutathione assay (Trevigen, Gaithersburg, MD, USA). For total GSH assay, liver tissue samples were mixed with ice-cold 5 % (w/v) metaphosphoric acid (Sigma-Aldrich) for homogenization, and then centrifuged at 12,000 *g* for 15 min at 4 °C. After serially diluting samples, we mixed prepared samples, 1X Assay Buffer, and 4 μM GSSG in 96-well plates. Reaction Mix (150-μl) was added to each well, and then the absorbance in the wells was tested at 405 nm using Sunrise ELISA Reader (TECAN, Männedorf, Switzerland). For oxidized GSH assay, prepared samples were mixed with 4-vinylpyridine (2 M, Sigma-Aldrich) and 4-μM GSSG. After incubating in a 96-well plate for 1 h at room temperature, we followed the protocols to measure total GSH.

Liver tissue samples (0.05 g) were homogenized with ice-cold 1X PBS containing heparin (0.16 mg/ml). The homogenates were centrifuged at 1500 *g* for 15 min at 4 °C. 4-HNE and MDA levels were measured using 4-HNE ELISA kit and MDA ELISA kit (MyBioSource, San Diego, CA, USA), according to manufacturer's instructions. The absorbance in the wells was measured at 450 nm using Sunrise ELISA Reader.

### Cell culture

The HSC-T6 and LX-2 cells were both generous gifts from Prof. S.L. Friedman (Mount Sinai Medical School, USA), the former being an immortalized cell line of rat HSCs and the latter, immortalized human HSCs. HSC-T6 and LX-2 cells were incubated in DMEM (Corning, Tewksbury, MA, USA) containing separately 10 % and 2 % fetal bovine serum (FBS, pH 7.0; Gibco BRL, Gaithersburg, MD, USA) at 37 °C in 5 % CO_2._

### Wound-healing assay

HSCs were seeded in culture inserts (ibidi, Martinsried, Germany) on collagen coated 24-well plates. After 24-h serum starvation, cells with cell-free zone were pre-treated with osthole (1, 3 and 10 μg/ml) for 1 h, then incubated with TGF-β1 for 24 h. HSCs migration was quantified by the area of cells in the cell-free zone, as previously reported by us [19].

### Trans-well invasion assay

Serum-starved HSCs were cultured on chemotaxis chamber Millicell® (Merck Millipore) with 8-μM pores, which were pre-coated with Matrigel^™^ Matrix (BD Biosciences, San Jose, CA, USA). The bottom wells were containing serum-free medium and TGF-β1 plus osthole (1, 3 and 10 μg/ml). The cells and chambers were incubated at 37 °C for 24 h. Cells migrated to the lower surface were stained with hematoxylin to define the cell nuclei, as previously reported by us [19].

### Luciferase assay

HSCs were transfected with Fugene-6 (Roche, Basel, Switzerland). NFκB-Luc reporter construct (1 μg/well) (Stratagene, La Jolla, CA, USA) was added to cells with plasmid CMV-β-galactosidase (CMV-β-gal, 0.2 μg/well; Promega, Madison, WI, USA). CMV-β-gal served as an internal control to normalize the transfection efficiency. After osthole (1, 3 and 10 μg/ml) treatment for 1 h, NF-κB activity was stimulated by TNF-α for 6 h. Cell lysates and luciferin (Promega) were detected by a luminometer-VICTOR2 Multilabel Counter (Perkin Elmer Inc., Waltham, MA, USA), as previously reported by us [19].

### Contraction assay

The protocol was modified from a previously published method [20]. In brief, the cells were mixed with 1-M NaOH, 10X Hank’s buffer, inducers ET-1 or TGF-β1, and type I collagen. Collagen gels were treated for 48 h with osthole (10 μg/ml) and the area of each gel was compared.

### Statistical analysis

The data are expressed as mean ± standard deviation. One-way analysis of variance (ANOVA, under the Tukey’s test) was used for comparison of molecular and biochemical parameters. A non-parametric method (the Dunn procedure under the Kruskal-Wallis test) was used for multiple pairwise comparisons between groups for the histological grades of fibrosis. The statistical significance test was done by Scheffe’s test to confirm where the differences occurred between groups. Software SPSS was used for statistic analysis. A significant difference was considered at *p* < 0.05.

## Results

### Osthole protected the rat liver against TAA-stimulated injury

**Fig. 1:**
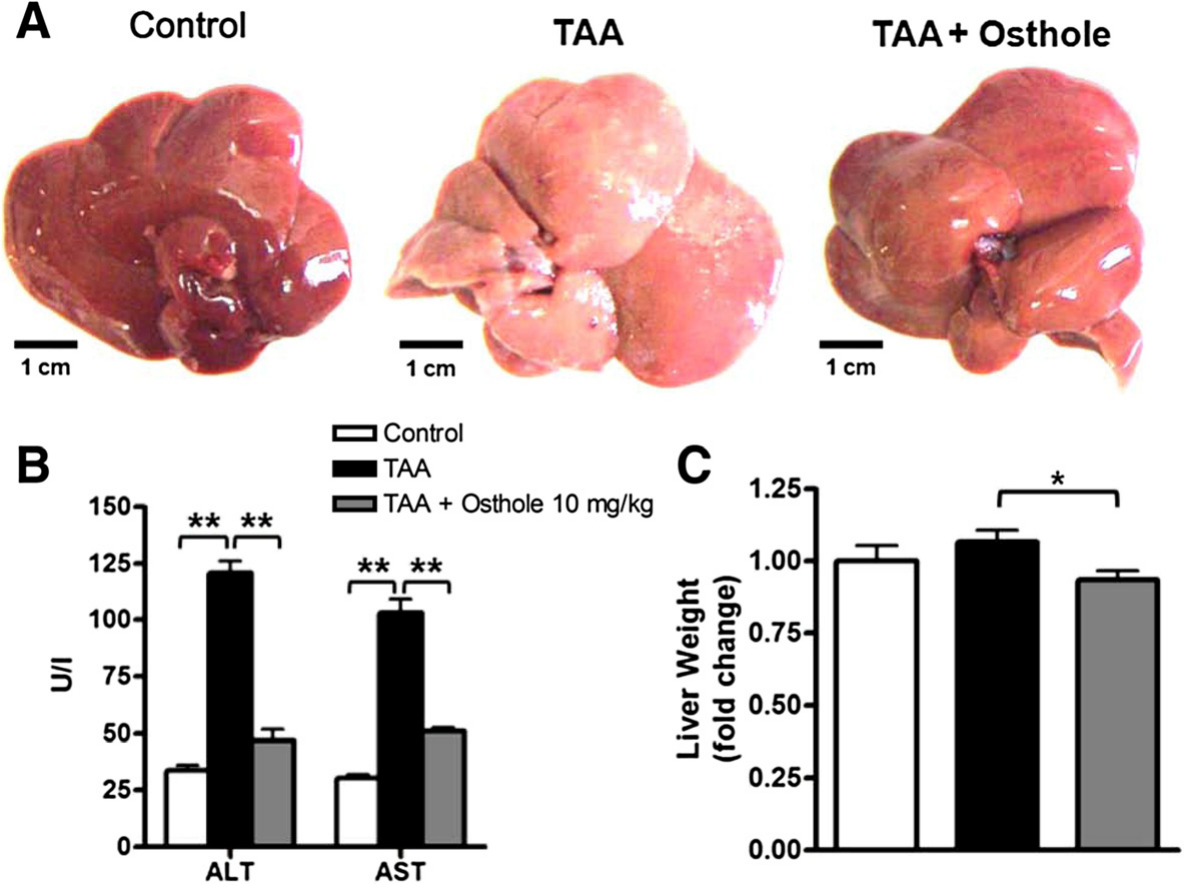
General profiles in control rats and TAA-induced fibrotic rats with or without osthole treatment. **a** Rat liver images from three groups: control rats were given only CMC (*n* = 8); and TAA-group rats were given CMC with TAA injection (*n* = 10); TAA + osthole-group rats were gavage osthole (10 mg/kg) in CMC with TAA injection (*n* = 10). The protocol of treatment was described in the Methods. Scale bar represents 1 cm for livers. **b** Plasma levels of ALT and AST from all groups. **c** Liver weight was recorded after sacrifice. Data are shown as mean ± SD of 8 rats in each group.*p < 0.05; **p < 0.01, compared with other groups

To identify the therapeutic effect of osthole in TAA rats, we first observed the liver condition of each group. In macroscopic views, control livers showed smooth surface and brown color, but TAA-group livers displayed pink and numerous irregular nodules. Livers from TAA + osthole rats exhibited normally dark red color without surface nodularity (Fig. 1a). The mortality among three groups was not significantly different (Additional file 3: Figure S2). We further assessed the effect of osthole treatment on liver injury by biochemical analyses of plasma enzymes. TAA-injected rats showed significantly higher ALT and AST activities than control rats, suggesting hepatic injury due to TAA. Results also showed that the group had lower levels of ALT and AST than the TAA group (Fig. 1b). There was no renal impairment according to the value of creatinine in all groups (data none shown). The body weight of TAA-treated rats was significantly lighter than that of control rats, and there was no difference in body weight between TAA-treated rats with and without osthole treatment (Additional file 4: Figure S3). There was no difference in liver weight between control and TAA rats. However, TAA + osthole-treated rats showed a significant decrease in liver weight compared with TAA rats (Fig. 1c), suggesting that osthole treatment diminished hepatic injury in TAA rats.

### Osthole attenuated TAA-induced rat liver fibrosis

**Fig. 2:**
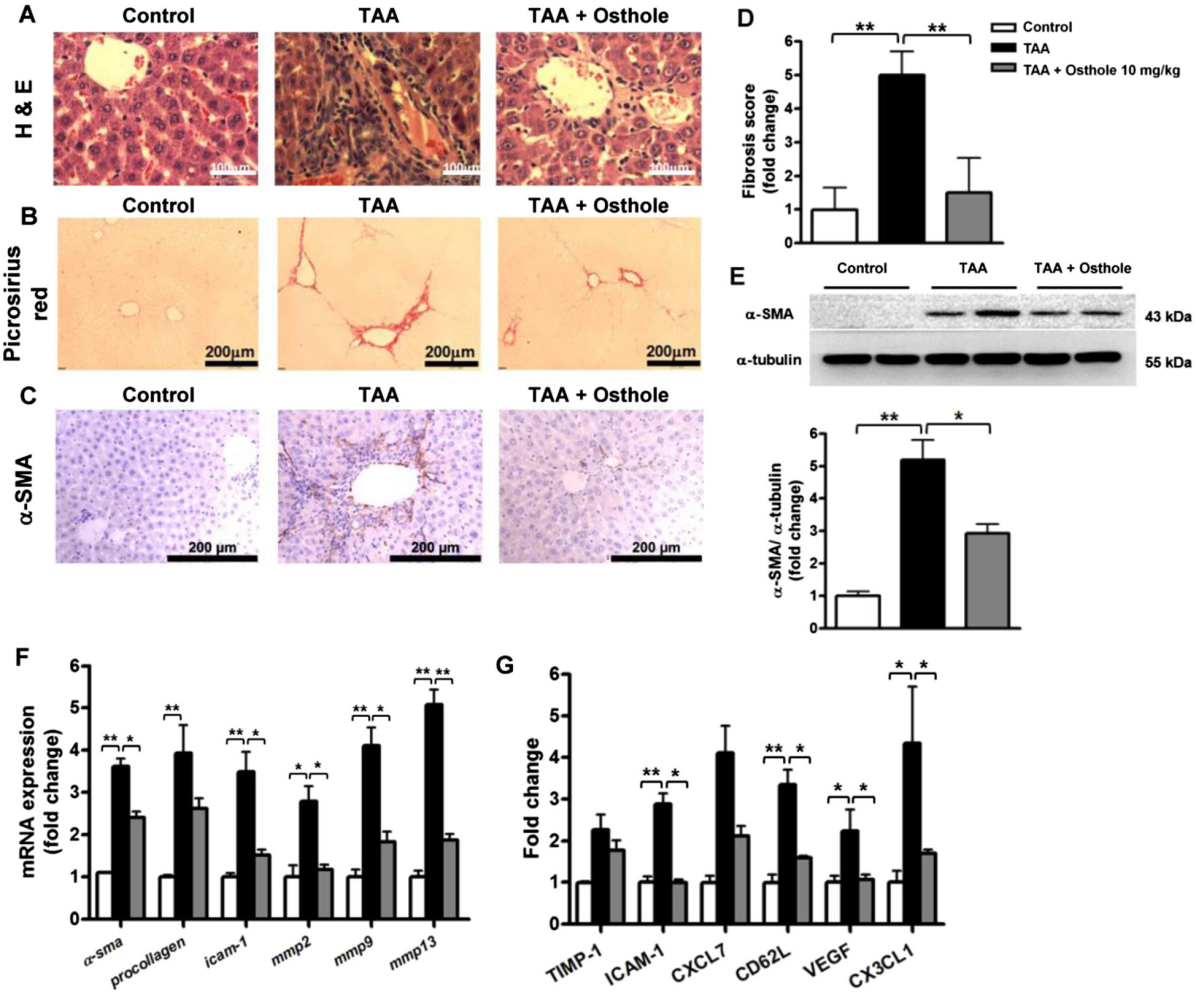
Osthole attenuated liver injury and fibrogenesis in the TAA rat model. Representative liver sections were obtained from groups as follows: control, TAA and osthole-treated TAA rats. Liver sections were analyzed with **a** H&E-, **b** Picrosirius red- and **c** immunostaining of α-SMA. **d** Fibrosis scores were assessed by a pathologist in a blind fashion. **e** Quantification of collagen-positive area was performed using Metamorph software. **f** α-SMA protein in liver tissues detected by Western blotting analysis. **g** The expressions of *α-sma* and *procollagen I*, *icam-1*, *mmp2*, *mmp9* and *mmp13* transcripts in livers measured by qRT-PCR. h ELISA of TIMP-1, ICAM-1, CXCL7, CD62L, VEGF, and CX3CL1 in plasma of various groups. Data are shown as mean ± SD of 8 rats in each group. *p < 0.05; **p < 0.01, compared with other groups

The function of osthole on TAA-induced hepatic injury and fibrosis were then evaluated based on the representative H&E- and Picrosirius red-stained liver sections. We observed changes in histological examination of livers from TAA rats compared with control rats, such as damage of hepatocytes and progressive increase and expansion of fibrous septa. Histological analysis revealed the suppressive effects of osthole in TAA-induced hepatic injury and fibrosis (Fig. 2a). Picrosirius red stain showed collagen fibres deposited in TAA-treated livers significantly compared with control rats (Fig. 2b). In addition, decreased α-SMA-positive cells were found in the fibrous septa portal tracts and sinusoids of the TAA + osthole-treated livers by immunohistochemical staining compared with TAA-treated livers (Fig. 2c). Consistently, the fibrosis score in TAA rats (0.72 ± 0.10) was significantly higher than that in control rats (0.14 ± 0.09), and osthole treatment (0.21 ± 0.14) significantly ameliorated hepatic fibrosis in TAA rats (Fig. 2d). The results showed that TAA intoxication led to a significant increase of collagen-positive area in the rat liver, which was attenuated by osthole treatment (Fig. 2e). We further confirmed that osthole treatment significantly attenuated TAA-induced α-SMA protein expression by Western blotting analysis (Fig. 2f). TAA-induced fibrosis-related gene expression, such as *α-sma* and *procollagen I*, was significantly reduced by osthole administration. There were also obvious decreases of mRNA levels in ECM synthesis*-*related genes*,* including *icam-1, mmp2, mmp9* and *mmp13* (Fig. 2g). To investigate whether osthole treatment affects the secretion of TAA-induced ECM-related cytokines and chemokines, we measured the production of distinct ECM-formation mediators by cytokine array. Results illustrated that osthole treatment exhibited a significant reduction of ICAM-1, CD62L, VEGF, and CX3CL1 levels. TIMP-1 and CXCL7 levels in the TAA + osthole group tended to be lower than those in the TAA group, but did not reach statistic significance (Fig. 2h). The data suggested that osthole treatment alleviated TAA-caused liver fibrosis and ECM synthesis.

### Osthole suppressed TAA-induced oxidation and inflammation in rat liver

**Fig. 3:**
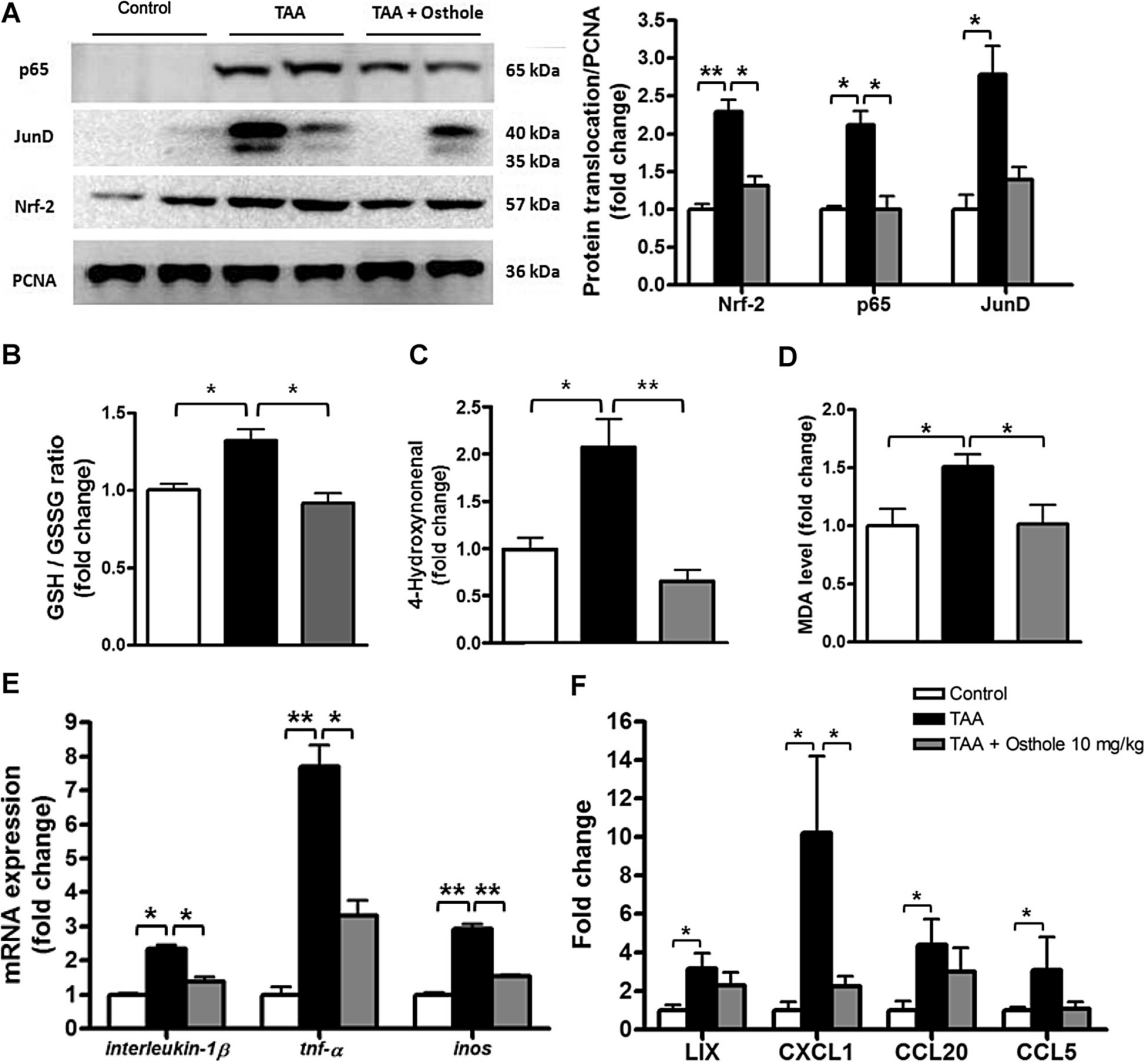
Osthole suppressed inflammation and oxidation in TAA-injected rat liver. **a** Nuclear fractions of rat liver from different treatment groups were analyzed by Western blotting analysis. For expression of p65, JunD and Nrf-2, PCNA expression served as loading control in nuclear protein. **b** GSH/GSSG ratio in the livers was measured by Glutathione kit. **c** Levels of 4-HNE in the livers were measured using 4-HNE ELISA kit. **d** Levels of MDA in the livers were measured using MDA ELISA kit **e** qRT-PCR showed the expressions of *interleukin-1β, tnf-α* and *inos* transcripts in rats. **f** ELISA of LIX, CXCL1, CCL20 and CCL5 in plasma. Data are shown as mean ± SD of 6 rats in each group. *p < 0.05; **p < 0.01, compared with other groups

In chronic liver diseases, inflammation is a major feature associated with fibrogenesis [21, 22]. We postulated that osthole treatment might attenuate TAA-induced injury by inhibiting liver inflammation. The H&E-stained liver sections in TAA injected rats showed portal triad inflammation and periportal inflammatory cell infiltration (Fig. 2a). We further prepared nuclear fractions to determine translocation of p65 and JunD. The p65 is the main subunit of NF-κB which can be triggered to translocate into the nucleus then activating transcription of various genes. JunD is a functional component of the activator protein-1 (AP-1) transcription factor complex known to activate AP-1-mediated transcription of genes in inflammatory response [23]. Data of nuclear fraction using Western blotting analysis indicated that there were more p65 and JunD nuclear translocation in TAA-injected livers than in the control group. Osthole treatment evidently reduced p65 translocation into nuclei in TAA-injected rats (Fig. 3a). The JunD translocation in osthole-treatment group tended to be lower than those in the TAA group. Nrf-2 plays a critical role in stress-inducible genes and also in cellular resistance to oxidants. TAA-promoted Nrf-2 translocation was profoundly down-regulated by osthole treatment, suggesting that oxidative stress in the liver might be counteracted by osthole (Fig. 3a). In Glutathione assay, we measured the levels of total GSH and oxidized GSSG, and then determined the level of GSH/GSSG ratio. The results showed that TAA treatment led to higher GSH/GSSG ratio, and osthole treatment restored GSH depletion in the liver (Fig. 3b). In addition, we found that osthole reduced the amount of TAA-caused lipid peroxidative products 4-HNE and MDA in the liver (Fig. 3c, d). To further explore the anti-inflammatory role of osthole treatment, the mRNA levels of *interleukin-1β, tnf-α* and *inos* genes in the rat liver were evaluated by qRT-PCR. The expression levels of these inflammation-related genes in the liver were increased in TAA-injected rats, which were obviously attenuated by osthole treatment (Fig. 3e). Additionally, levels of inflammation-related chemokines in the TAA group were significantly higher than those in control rats, but only CXCL1 levels showed significant reduction after osthole treatment. LIX and CCL20 levels in the TAA + osthole group tended to be lower than those in the TAA group, but did not reach statistic significance (Fig. 3f).

### Osthole downregulated chemotaxis and contraction of activated HSCs

**Fig. 4:**
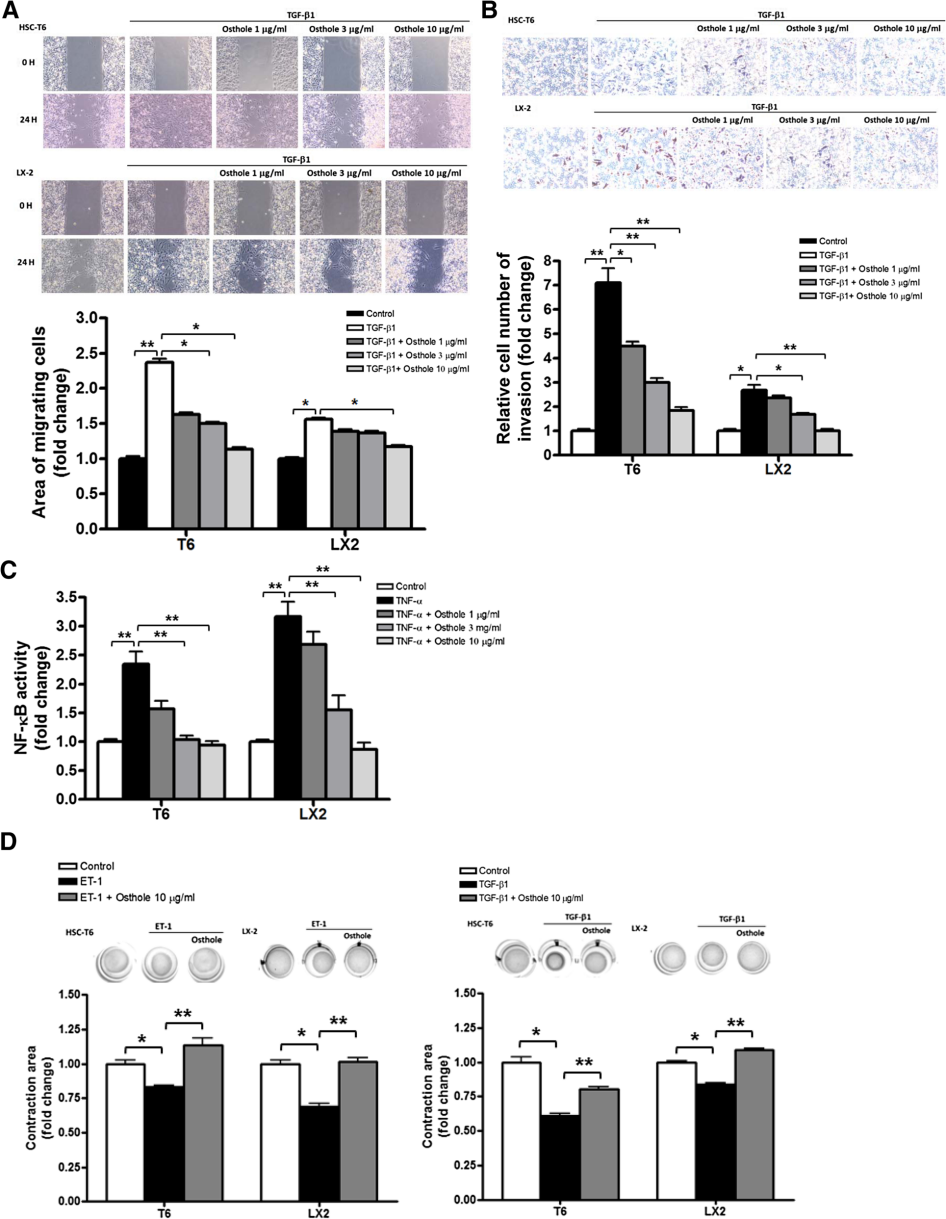
Osthole downregulated HSC activation. **a** Wound healing assay in HSCs (both HSC-T6 and LX2 cells). Cells were treated by osthole (1, 3 and 10 μg/ml) after creating wounds. Graphs represent cell migration assessed at 0 h and 24 h after exposure to TGF-β1 (1 ng/ml), and quantification of wound closure was shown below, *n = 3*. **b** Cell invasion assay in HSCs. Cells were treated by osthole (1, 3 and 10 μg/ml) and then stimulated with TGF-β1 for 24 h. Graphs displaying the bottom side of the filter inserts with cells that migrated through the filter pores. Graphs of quantification graphs represent the analysis of the cell count. **c** NF-κB activity of HSCs which were transfected with CMV-βgal and the reporter plasmid containing NF-κB responsive region for 24 h. Cells were pre-treated with osthole (1, 3 and 10 μg/ml) for 1 h, followed by 6 h TNF-α (1 ng/ml) stimulation, then NF-κB activity was detected by luminescence. CMV-βgal was used as internal control to normalize the transfection efficiency. *n = 3*. **d** Osthole-inhibited ET-1- or TGF-β1-induced HSC contraction. Cultured HSCs were serum-starved 24 h prior to seeding onto collagen lattices. Cells were pre-treated with control or osthole in 10 μg/ml and subsequently treated with ET-1 (1 nmol/l) or TGF-β1(1 ng/ml) after 30 min. Graphs of quantification graphs represent the analysis of the area of collagen circle. *n = 3*. *p < 0.05; **p < 0.01, compared with other groups

The *in vivo* results above suggested that osthole could suppress TAA-caused hepatic fibrogenesis and inflammation in rats. HSCs play an important role during hepatic fibrogenesis, and HSC activation is also crucially related to hepatic inflammation. Osthole (1, 3, and 10 μg/ml) showed no cytotoxicity to HSC-T6 and LX-2 cells after 24 h treatment (Additional file 5: Figure S4). We utilized HSC-T6 and LX-2 to determine whether osthole could inhibit HSC activation induced by chemotactic stimulation. Using wound-healing assays, the inhibitory effect of osthole on TGF-β1-induced HSC migration were revealed. After 24 h exposure to TGF-β1, HSCs (both HSC-T6 and LX-2) showed obvious migration to the cell-free zone. Moreover, osthole (10 μg/ml) inhibited TGF-β1-induced migration in HSCs significantly (Fig. 4a). We also performed trans-well invasion assay to assess the ability of cell invasion. Osthole (1-10 μg/ml) curbed HSC invasion in response to TGF-β1. The cell number of TGF-β1-stimulated HSCs was higher than that in the control group, whereas osthole treatment (3, 10 μg/ml) decreased TGF-β1-stimulated invasion significantly in both HSC cell lines (Fig. 4b). Furthermore, we used the luciferase assay to determine NF-κB activity, which is a hallmark of HSC activation. In both HSC cell lines, osthole (3, 10 μg/ml) led to a marked inhibition of TNF-α-induced NF-κB luciferase activity (Fig. 4c). HSC activation exhibits many features, the most prevailing of which includes tissue contraction mediated by contractile myofibroblasts. Therefore, we measured HSC contractility by ET-1 and TGF-β1 stimulation, both well known to induce HSC contraction in the liver. We treated HSCs plated on collagen lattices with osthole (10 μg/ml) and ET-1 or TGF-β1 for 48 h. Osthole significantly reduced both ET-1- and TGF-β1-promoted HSC contraction (Fig. 4d). We substantiated our hypothesis that osthole was capable of interrupting HSC activation in HSC-T6 and LX-2 cells.

## Discussion

In the present study, we demonstrated that osthole showed anti-hepato-fibrotic effects on TAA-caused injured livers with attenuating (a) hepatic fibrosis score and collagen deposition, (b) hepatic α-SMA protein accumulation, (c) hepatic mRNA expressions of *α-sma, procollagen I*, *icam-1, mmp2, mmp9* and *mmp13,* (d) hepatic translocation of p65, JunD, and Nrf-2 proteins, (e) plasma levels of ALT and AST, (f) plasma levels of several inflammation- and fibrosis-related cytokines and chemokines. In our further *in vitro* study of HSCs, osthole exhibited suppressive effects on HSC activation, such as inhibiting TGF-β1-induced migration and invasion, TNF-α-stimulated NF-κB activity, and TGF-β1- or ET-1-stimulated contractility. Overall, the pure compound osthole from *Cnidium monnieri* (L.) Cusson significantly suppressed hepatic fibrosis in TAA-injected rats, and inhibited HSCs activation, which is the key event associated with hepatic fibrosis.

TAA causes toxicity in hepatocytes and then induces acute and chronic liver injury through free radical and oxidative stress [24]. The first step of TAA metabolism is through flavin-adenin dimucleotide (FAD)-containing monooxygenase (FMO) system in mammals [25]. Next, the resulting TAA metabolites would be converted to TAA-S-dioxide by cytochrome P450 monoxygenases [26]. TAA-S-dioxide could covalently bind to the molecules in the liver and trigger oxidative stress to cells [27, 28]. The serial events would eventually lead to liver necrosis. Previous studies indicated that the mechanism of TAA-caused liver injury involves the induction of the oxidative stress in multiple cell types. Researchers have found that both catalase and glutathinone peroxidase can reduce the TAA-induced free radical production [24, 29]. The depletion of the antioxidant GSH is one of the indices of oxidative stress. High reduced GSH levels are associated with increased oxidative damage by free radicals in the TAA-group. GSH reductase converts GSSG to reduced GSH. We have found that osthole restored the levels of GSH/GSSG. Moreover, the peroxidative products 4-HNE and MDA induced by TAA-injection were both decreased by osthole treatment significantly. These results indicated that osthole ameliorated TAA-induced injury through reducing cellular oxidation.

We used 10 μg/ml as the maximum concentration of osthole in preliminary *in vitro* assays. Therefore, we estimated that around 10 mg⁄kg is needed *in vivo* as a dose of osthole, and we also used 10 μg/ml to investigate more suppressive effects of osthole in HSC activation in present *in vitro* assays. In addition, we referenced study which utilized 10 mg⁄kg (i.v.) in rats for pharmacokinetics experiments [30]. Although our gavage method might result in lower bioactivity, present results showed that osthole at 10 mg⁄kg could improve TAA-caused hepatic fibrosis and inflammation in rats.

Osthole attenuated hepatic fibrosis scores and accumulation of α-SMA protein and collagen, together with plasma levels of ALT and AST in TAA-injected rats. However, it did not improve body weight loss. These consequences suggested that there might be only partial amelioration with osthole treatment of pathological state in TAA-caused lesion. Osthole is an active constituent of Chinese medicine-*Cnidium monnier* (L.) Cusson [31], which has been used clinically in traditional prescriptions such as “She Chuang Zi” for anti-allergic action [32], androgenic action [33]. anti-inflammation [34] and anti-apoptosis [35]. Modern reports have shown that osthole possesses therapeutic function in various diseases, such as alleviating skin inflammation [36], protecting lung [37] and kidney injuries [38], anti-diabetic [39, 40] and anti-osteoporosis [41]. In the hepatic field, osthole has been shown to inhibit the secretion of HBV surface antigens *in vitro* [13] and shown to prevent HCC cells growth [42, 43]. There were several studies focused on the function of osthole in liver steatosis [9, 10, 44], but few discussion about its efficacy in hepatic fibrosis. We demonstrated that osthole did ameliorate hepatic inflammation and fibrogenesis in TAA-model.

NF-κB is a major ubiquitous transcription factor regulating the expression of genes involved in inflammatory responses. Sensitizing NF-κB signaling via activation of IκB-kinase (IKK) complex and degradation of IκBα protein, in turn, releases the cytosolic heterodimer p65/p50, and then p65 translocates into nucleus to activate transcription of various genes. Previous studies mentioned that osthole suppresses the activity and protein expression of NF-κB [11, 42]. Our findings also indicated osthole inhibited p65 translocation *in vivo*, and blunted *in vitro* NF-κB activity in HSCs. JunD is the member of the JUN family associated with proinflammatory transcription factor AP-1 [45] and it is important for cytokine-induced liver damage [46]. Although there is no evidence about the cell-specific function of JunD, we could find positive relevant between p65 and JunD in osthole-attenuated TAA-caused liver damage. Our data showed that TAA injections induced Nrf-2 translocation in injured liver, and osthole treatment ameliorated this phenomenon. There was a study showing that deletion of Nrf-2 may abolish liver injury [47]. Alternatively, osthole has potential to reduce cellular oxidation and hence oxidation-induced Nfr-2 activation is reduced (Fig. 3a), which may counteract Nrf-2-induced responses. Evidence has shown that osthole induces gene expressions of proinflammatory cytokines such as TNF-α and IL-2 [48]. Moreover, osthole inhibits TNF-α production and showed anti-oxidative ability in alcohol-induced fatty liver in mice [10]. In our study, we found that osthole treatment downregulated TAA-induced mRNA levels of *tnf-α, interleukin-1β*, and *inos*. Reports have shown CXCL1 is genetic risk factors involved in the pathogenesis of NASH and alcoholic cirrhosis, and CXCL1 is a ligand for CXC chemokine receptor family expressed on HSCs [49, 50]. In our cytokine assay, results showed that production of CXCL1 was reduced in osthole-treated TAA-rats. Our data indicated that osthole exerted inhibitory effects on inflammatory responses in hepatic fibrosis.

Inflammation and HSC activation could be considered as two distinct mechanisms in fibrogenesis. However, both processes affect each other in several ways [2, 3]. When the liver is injured by multiple threatening, the damaged liver tissue is surrounded by myofibroblasts and inflammatory cells. In our present study, results indicated that osthole decreased the infiltration of multiple cell types in the rat liver (Fig. 2a). It was reported previously that at the early stage of liver injury, infiltrated inflammatory cells around injured sites can secret cytokines and chemokines to induce HSCs activation [51]. In this study, we observed that osthole reduced the hepatic production of α-SMA, inflammatory cytokines and chemokines in TAA-treated rats. Our *in vitro* results also indicated that osthole inhibited both TNF-α-induced NF-κB activity and TGF-β-induced α-SMA in HSCs*.* Therefore*, in vivo* and *in vitro* results demonstrated that osthole inhibited both HSC activation and inflammation in rat livers.

Chemotaxis is one of features in activated HSCs, and this action in normal physiology serves to repair injured tissues. Whereas, moving HSCs keep secreting inflammatory mediators and producing ECM once the liver starts unhalted fibrogenic processes. We performed two methods to confirm that osthole inhibited TGF-β1-induced wound-healing chemotaxis and invasion of HSCs. There was a report showing the inhibitory function of osthole in migration and invasion of breast cancer cells [52]. Chemokine secretion has been known for inducing cell migration [53]. In our *in vivo* study, we found osthole treatment reduced chemotaxis-related cytokines. Cell contractility is a major characteristic of HSC activation as well. Activated HSCs contribute to increased portal pressure during progression from hepatic fibrosis to cirrhosis and HSCs are reported as a main target for ET-1 action in liver [54]. Briefly, ET-1 promotes HSCs to show higher contractility and then increases portal venous pressure. HSC activation mainly requires TGF-β1 which provokes diverse inflammatory and fibrogenic responses [55]. To our knowledge, there has been no scientific report on whether osthole can affect HSC contractility, and our current results show that osthole suppressed TGF-β1- or ET-1-stimulated HSC contraction.

## Conclusion

Osthole could be considered as a potential therapeutic agent in view of our current pharmacological results. In conclusion, our results demonstrated that osthole ameliorated TAA-injected liver injury and fibrogenesis in rats, possibly through inhibiting HSC activation.

## Additional files

Additional file 1: Figure S1. Chemical structure of osthole. (TIFF 68 kb) Additional file 2: Table S1. The sequences of primers used in this study. (TIFF 2369 kb) Additional file 3: Figure S2. The effect of osthole on survival of TAA-induced fibrotic rats. Survival rate of the animals treated with TAA (longer dotted line), TAA with osthole treatment (dashed line) and control (solid line). The data were analyzed using the Kaplan-Meier method. (TIFF 516 kb) Additional file 4: Figure S3. The effect of osthole on body weight of TAA-treated rats. Body weight was recorded twice a week. There was a significant decrease in TAA rats than control, but no statistic difference between TAA rats and osthole-treated TAA rats in body weight. (TIFF 391 kb) Additional file 5: Figure S4. Cell viability of osthole in HSC-T6 and LX-2 cells. There was no cytotoxicity of osthole (1, 3, and 10 μg/ml) in HSC-T6 and LX-2 cells for 24 h. (TIFF 746 kb)

## Abbreviations

ALT: alanine transaminase
AP-1: activator protein-1
α-SMA: α-smooth muscle actin
AST: aspartate transaminase
CMC: carboxyl methyl cellulose
ECM: extracellular matrix
ET-1: endothelin
GSH: glutathione
GSSG: oxidized glutathione
4-HNE: 4-Hydroxynonenal
H&E: Hematoxylin-eosin
HSC: hepatic stellate cell
i.p.: intraperitoneal
ICAM-1: intracellular adhesion molecule-1
i.v.: intravenous injection
IKK: IκB-kinase
MDA: malondialdehyde
Nrf-2: nuclear factor erythroid-derived 2
qRT-PCR: quantitative reverse transcription-polymerase chain reaction
SD: Sprague–Dawley
TAA: thioacetamide
TGF-β1: tumor growth factor-β1
TIMP-1: tissue inhibitor of metallopeptidase-1
TNF-α: tumor necrosis factor-α

## References

[1] Hernandez-Gea V, Friedman SL (2011). Pathogenesis of liver fibrosis. Annu Rev Pathol..

[2] Schuppan D, Kim YO (2013). Evolving therapies for liver fibrosis. J Clin Invest.

[3] Friedman SL (2010). Evolving challenges in hepatic fibrosis. Nat Rev Gastroenterol Hepatol.

[4] Troeger JS, Mederacke I, Gwak GY, Dapito DH, Mu X, Hsu CC (2012). Deactivation of hepatic stellate cells during liver fibrosis resolution in mice. Gastroenterology.

[5] Brenner DA (2009). Molecular Pathogenesis of Liver Fibrosis. Trans Am Clin Climatol Assoc..

[6] Friedman SL (2012). Fibrogenic cell reversion underlies fibrosis regression in liver. Proc Natl Acad Sci USA.

[7] Kisseleva T, Cong M, Paik Y, Scholten D, Jiang C, Benner C (2012). Myofibroblasts revert to an inactive phenotype during regression of liver fibrosis. Proc Natl Acad Sci U S A.

[8] Basnet P, Yasuda I, Kumagai N, Tohda C, Nojima H, Kuraishi Y (2001). Inhibition of itch-scratch response by fruits of Cnidium monnieri in mice. Biol Pharm Bull.

[9] Zhang Y, Xie M, Xue J, Gu Z (2007). Osthole improves fat milk-induced fatty liver in rats: modulation of hepatic PPAR-alpha/gamma-mediated lipogenic gene expression. Planta Med.

[10] Sun F, Xie ML, Zhu LJ, Xue J, Gu ZL (2009). Inhibitory effect of osthole on alcohol-induced fatty liver in mice. Dig Liver Dis.

[11] Zhao X, Xue J, Wang XL, Zhang Y, Deng M, Xie ML (2014). Involvement of hepatic peroxisome proliferator-activated receptor alpha/gamma in the therapeutic effect of osthole on high-fat and high-sucrose-induced steatohepatitis in rats. Int Immunopharmacol.

[12] Sun F, Xie ML, Xue J, Wang HB (2010). Osthol regulates hepatic PPAR alpha-mediated lipogenic gene expression in alcoholic fatty liver murine. Phytomedicine.

[13] Huang RL, Chen CC, Huang YL, Hsieh DJ, Hu CP, Chen CF (1996). Osthole increases glycosylation of hepatitis B surface antigen and suppresses the secretion of hepatitis B virus in vitro. Hepatology.

[14] Dashti H, Jeppsson B, Hagerstrand I, Hultberg B, Srinivas U, Abdulla M (1989). Thioacetamide- and carbon tetrachloride-induced liver cirrhosis. Eur Surg Res.

[15] Muller A, Machnik F, Zimmermann T, Schubert H (1988). Thioacetamide-induced cirrhosis-like liver lesions in rats--usefulness and reliability of this animal model. Exp Pathol.

[16] Torres MI, Fernandez MI, Gil A, Rios A (1998). Dietary nucleotides have cytoprotective properties in rat liver damaged by thioacetamide. Life Sci.

[17] Hsu YC, Chiu YT, Cheng CC, Wu CF, Lin YL, Huang YT (2007). Antifibrotic effects of tetrandrine on hepatic stellate cells and rats with liver fibrosis. J Gastroenterol Hepatol.

[18] Desmet VJ, Knodell RG, Ishak KG, Black WC, Chen TS, Craig R (2003). Formulation and application of a numerical scoring system for assessing histological activity in asymptomatic chronic active hepatitis. J Hepatol.

[19] Liu YW, Huang YT (2014). Inhibitory effect of tanshinone IIA on rat hepatic stellate cells. PLoS One.

[20] Ngo P, Ramalingam P, Phillips JA, Furuta GT (2006). Collagen gel contraction assay. Methods Mol Biol..

[21] Marra F (2002). Chemokines in liver inflammation and fibrosis. Front Biosci..

[22] Friedman SL (2008). Mechanisms of hepatic fibrogenesis. Gastroenterology..

[23] Robison AJ, Nestler EJ (2011). Transcriptional and epigenetic mechanisms of addiction. Nat Rev Neurosci.

[24] Oliver JR, Jiang S, Cherian MG (2006). Augmented hepatic injury followed by impaired regeneration in metallothionein-I/II knockout mice after treatment with thioacetamide. Toxicol Appl Pharmacol.

[25] Chieli E, Malvaldi G (1984). Role of the microsomal FAD-containing monooxygenase in the liver toxicity of thioacetamide S-oxide. Toxicology.

[26] Chilakapati J, Korrapati MC, Shankar K, Hill RA, Warbritton A, Latendresse JR (2007). Role of CYP2E1 and saturation kinetics in the bioactivation of thioacetamide: Effects of diet restriction and phenobarbital. Toxicol Appl Pharmacol.

[27] Hunter AL, Holscher MA, Neal RA (1977). Thioacetamide-induced hepatic necrosis. I. Involvement of the mixed-function oxidase enzyme system. J Pharmacol Exp Ther.

[28] Neal RA, Halpert J (1982). Toxicology of thiono-sulfur compounds. Annu Rev Pharmacol Toxicol..

[29] Low TY, Leow CK, Salto-Tellez M, Chung MC (2004). A proteomic analysis of thioacetamide-induced hepatotoxicity and cirrhosis in rat livers. Proteomics.

[30] Tsai TH, Tsai TR, Chen CC, Chen CF (1996). Pharmacokinetics of osthole in rat plasma using high-performance liquid chromatography. J Pharm Biomed Anal.

[31] Chou SY, Hsu CS, Wang KT, Wang MC, Wang CC (2007). Antitumor effects of Osthol from Cnidium monnieri: an in vitro and in vivo study. Phytother Res.

[32] Matsuda H, Tomohiro N, Ido Y, Kubo M (2002). Anti-allergic effects of cnidii monnieri fructus (dried fruits of Cnidium monnieri) and its major component, osthol. Biol Pharm Bull.

[33] Yamahara J, Kozuka M, Sawada T, Fujimura H, Nakano K, Tomimatsu T (1985). Biologically active principles of crude drugs. Anti-allergic principles in "Cnidii monnieri". Chem Pharm Bull (Tokyo).

[34] Liu J, Zhang W, Zhou L, Wang X, Lian Q (2005). Anti-inflammatory effect and mechanism of osthole in rats. Zhong Yao Cai.

[35] Okamoto T, Kawasaki T, Hino O (2003). Osthole prevents anti-Fas antibody-induced hepatitis in mice by affecting the caspase-3-mediated apoptotic pathway. Biochem Pharmacol.

[36] Chen YF, Tsai HY, Wu TS (1995). Anti-inflammatory and analgesic activities from roots of Angelica pubescens. Planta Med.

[37] Shi Y, Zhang B, Chen XJ, Xu DQ, Wang YX, Dong HY (2013). Osthole protects lipopolysaccharide-induced acute lung injury in mice by preventing down-regulation of angiotensin-converting enzyme 2. Eur J Pharm Sci.

[38] Zheng Y, Lu M, Ma L, Zhang S, Qiu M, Wang Y (2013). Osthole ameliorates renal ischemia-reperfusion injury in rats. J Surg Res.

[39] Nam HH, Jun DW, Jeon HJ, Lee JS, Saeed WK, Kim EK (2014). Osthol attenuates hepatic steatosis via decreased triglyceride synthesis not by insulin resistance. World J Gastroenterol.

[40] Lee WH, Lin RJ, Lin SY, Chen YC, Lin HM, Liang YC (2011). Osthole enhances glucose uptake through activation of AMP-activated protein kinase in skeletal muscle cells. J Agric Food Chem.

[41] Ming LG, Zhou J, Cheng GZ, Ma HP, Chen KM (2011). Osthol, a coumarin isolated from common cnidium fruit, enhances the differentiation and maturation of osteoblasts in vitro. Pharmacology.

[42] Zhang L, Jiang G, Yao F, He Y, Liang G, Zhang Y (2012). Growth inhibition and apoptosis induced by osthole, a natural coumarin, in hepatocellular carcinoma. PLoS One.

[43] Lin VC, Chou CH, Lin YC, Lin JN, Yu CC, Tang CH (2010). Osthole suppresses fatty acid synthase expression in HER2-overexpressing breast cancer cells through modulating Akt/mTOR pathway. J Agric Food Chem.

[44] Song F, Xie ML, Zhu LJ, Zhang KP, Xue J, Gu ZL (2006). Experimental study of osthole on treatment of hyperlipidemic and alcoholic fatty liver in animals. World J Gastroenterol.

[45] Thomsen MK, Bakiri L, Hasenfuss SC, Hamacher R, Martinez L, Wagner EF (2013). JUNB/AP-1 controls IFN-gamma during inflammatory liver disease. J Clin Invest.

[46] Weitzman JB, Fiette L, Matsuo K, Yaniv M (2000). JunD protects cells from p53-dependent senescence and apoptosis. Mol Cell.

[47] Ni HM, Woolbright BL, Williams J, Copple B, Cui W, Luyendyk JP (2014). Nrf2 promotes the development of fibrosis and tumorigenesis in mice with defective hepatic autophagy. J Hepatol.

[48] Okamoto T, Kajino K, Hino O (2001). Hepatoprotective drugs for the treatment of virus-induced chronic hepatitis: from hypercarcinogenic state to hypocarcinogenic state. Jpn J Pharmacol.

[49] Talukdar S, Oh da Y, Bandyopadhyay G, Li D, Xu J, McNelis J (2012). Neutrophils mediate insulin resistance in mice fed a high-fat diet through secreted elastase. Nat Med.

[50] Nischalke HD, Berger C, Lutz P, Langhans B, Wolter F, Eisenhardt M (2013). Influence of the CXCL1 rs4074 A allele on alcohol induced cirrhosis and HCC in patients of European descent. PLoS One.

[51] Seki E, Brenner DA (2015). Recent advancement of molecular mechanisms of liver fibrosis. J Hepatobiliary Pancreat Sci.

[52] Yang D, Gu T, Wang T, Tang Q, Ma C (2010). Effects of osthole on migration and invasion in breast cancer cells. Biosci Biotechnol Biochem.

[53] Huang C, Jacobson K, Schaller MD (2004). MAP kinases and cell migration. J Cell Sci.

[54] Bataller R, Gines P, Nicolas JM, Gorbig MN, Garcia-Ramallo E, Gasull X (2000). Angiotensin II induces contraction and proliferation of human hepatic stellate cells. Gastroenterology.

[55] Seki E, De Minicis S, Osterreicher CH, Kluwe J, Osawa Y, Brenner DA (2007). TLR4 enhances TGF-beta signaling and hepatic fibrosis. Nat Med.

