# Supplementary figures and images for: Osthole ameliorates hepatic fibrosis and inhibits hepatic stellate cell activation
Source: J Biomed Sci. 2015 Aug 1;22(1):63. doi: 10.1186/s12929-015-0168-5 (PMC4522080; doi:10.1186/s12929-015-0168-5)

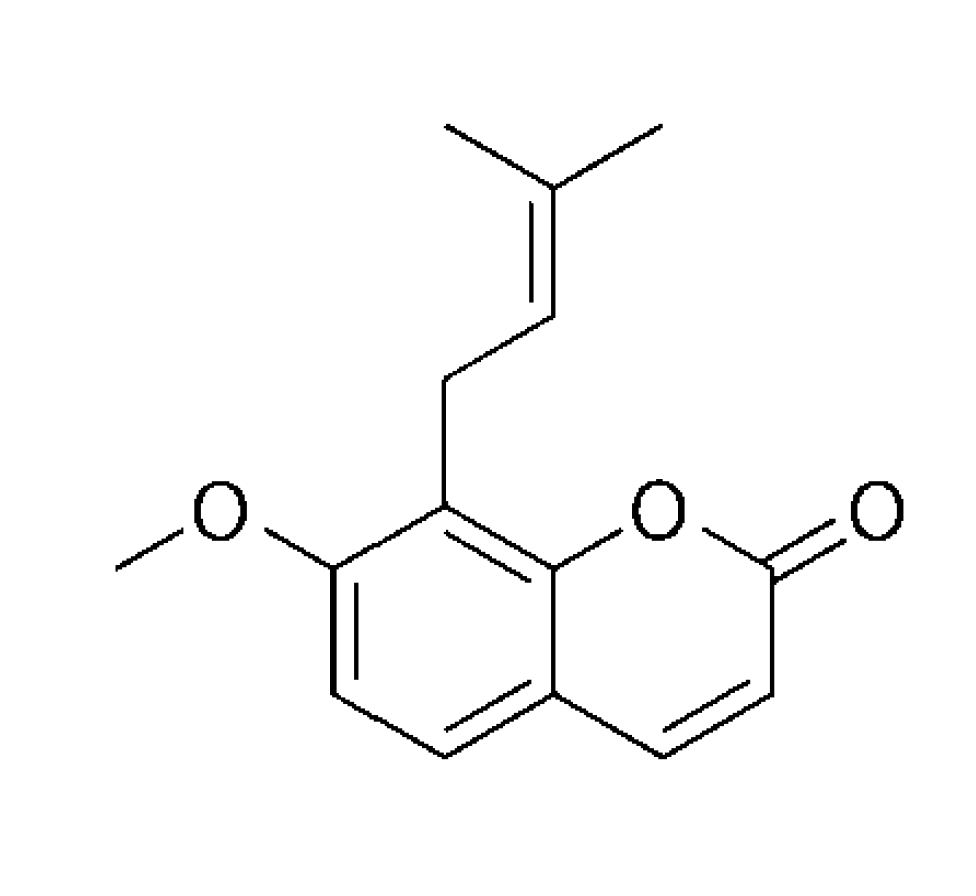

Supplement: Additional file 1: Figure S1. — Chemical structure of osthole. (TIFF 68 kb) [file 12929_2015_168_MOESM1_ESM.tiff]

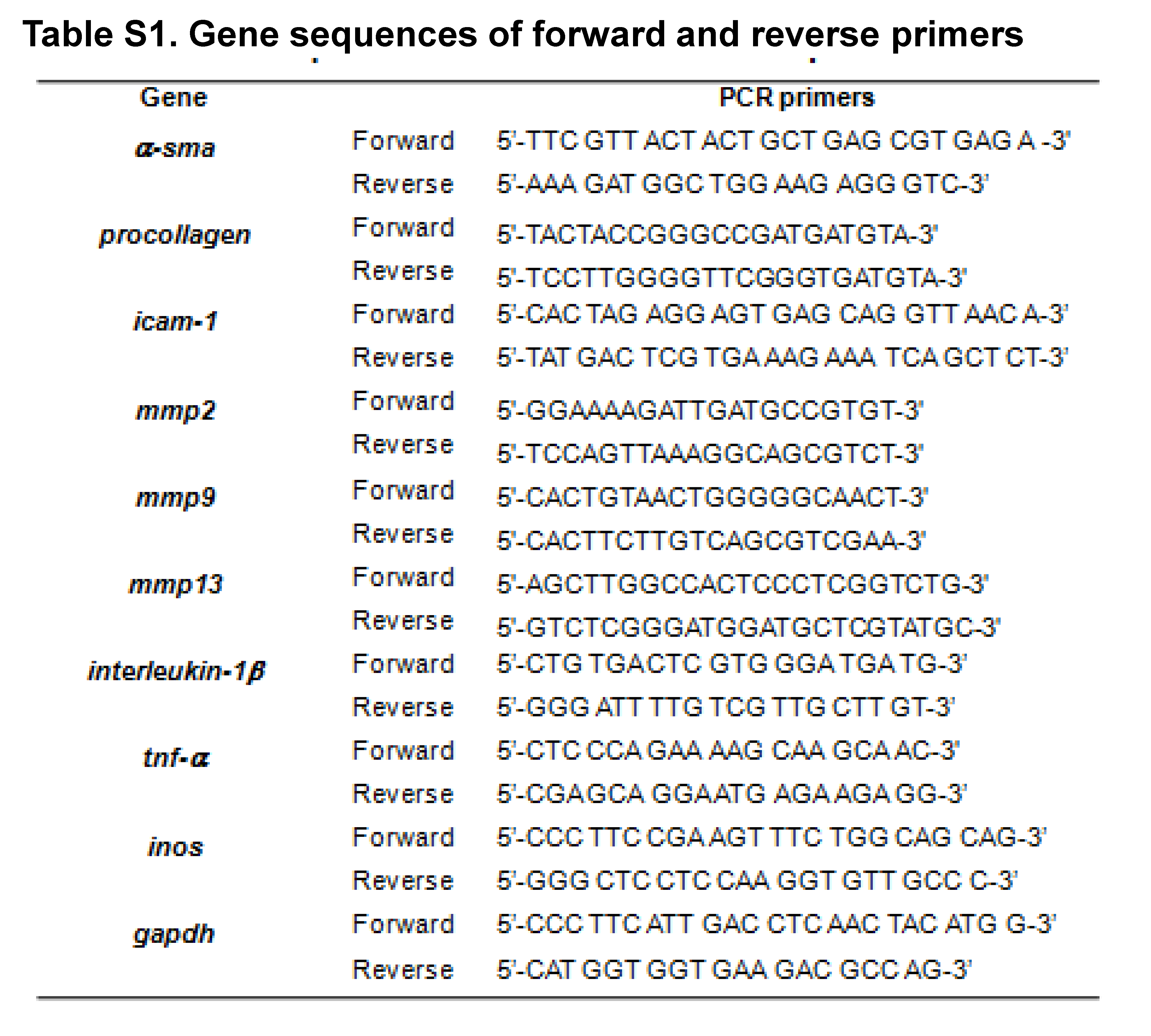

Supplement: Additional file 2: Table S1. — The sequences of primers used in this study. (TIFF 2369 kb) [file 12929_2015_168_MOESM2_ESM.tiff]

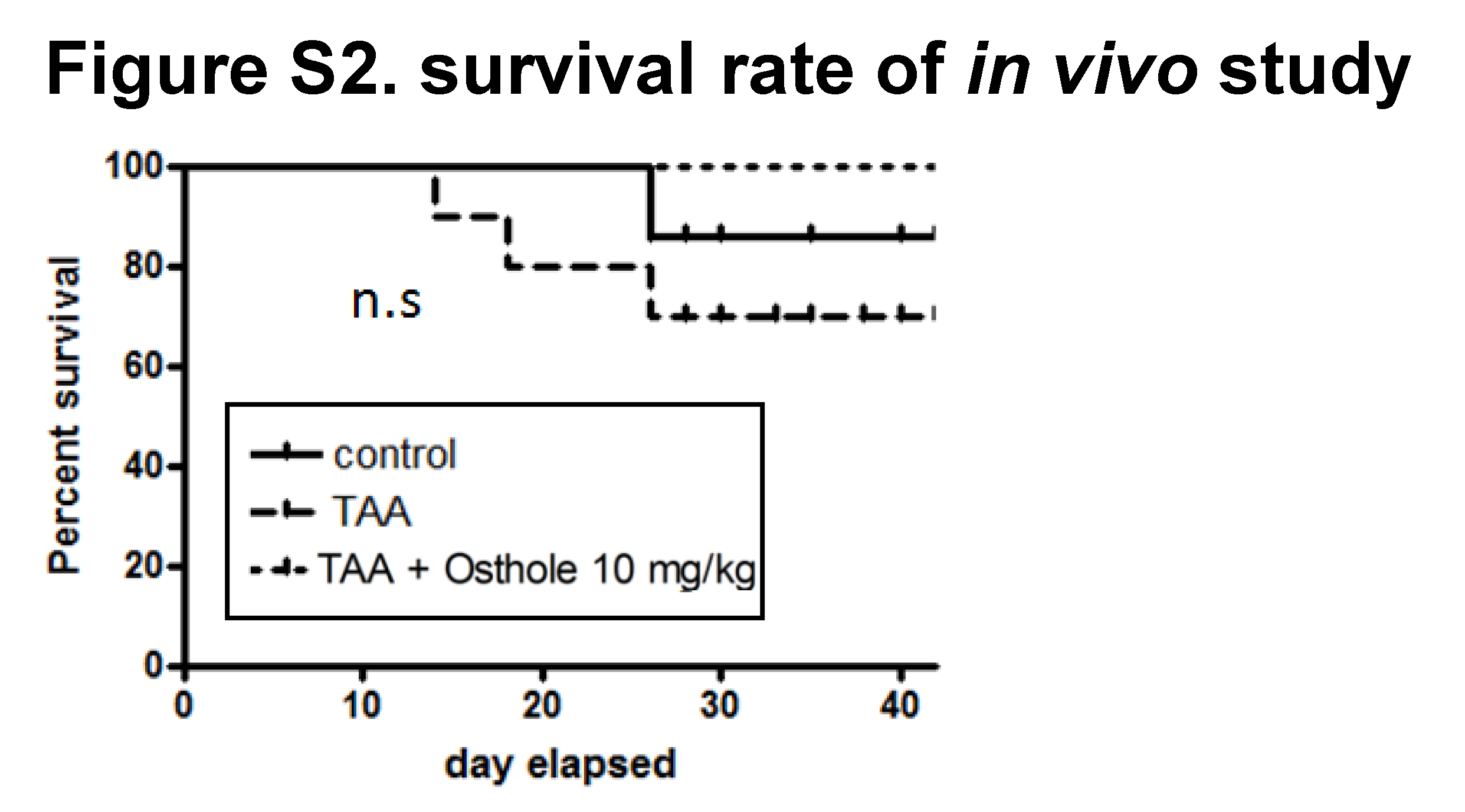

Supplement: Additional file 3: Figure S2. — The effect of osthole on survival of TAA-induced fibrotic rats. Survival rate of the animals treated with TAA (longer dotted line), TAA with osthole treatment (dashed line) and control (solid line). The data were analyzed using the Kaplan-Meier method. (TIFF 516 kb) [file 12929_2015_168_MOESM3_ESM.tiff]

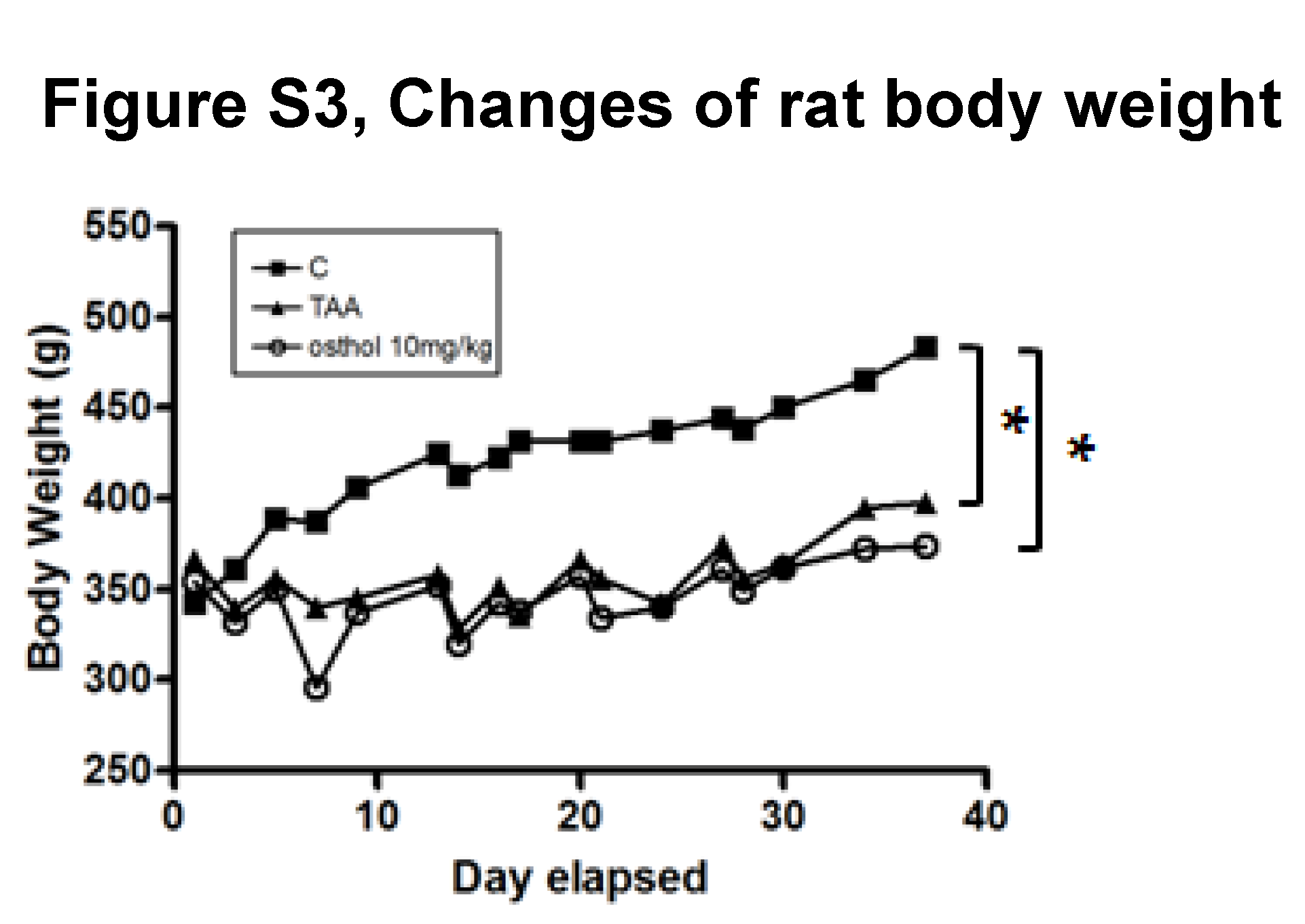

Supplement: Additional file 4: Figure S3. — The effect of osthole on body weight of TAA-treated rats. Body weight was recorded twice a week. There was a significant decrease in TAA rats than control, but no statistic difference between TAA rats and osthole-treated TAA rats in body weight. (TIFF 391 kb) [file 12929_2015_168_MOESM4_ESM.tiff]

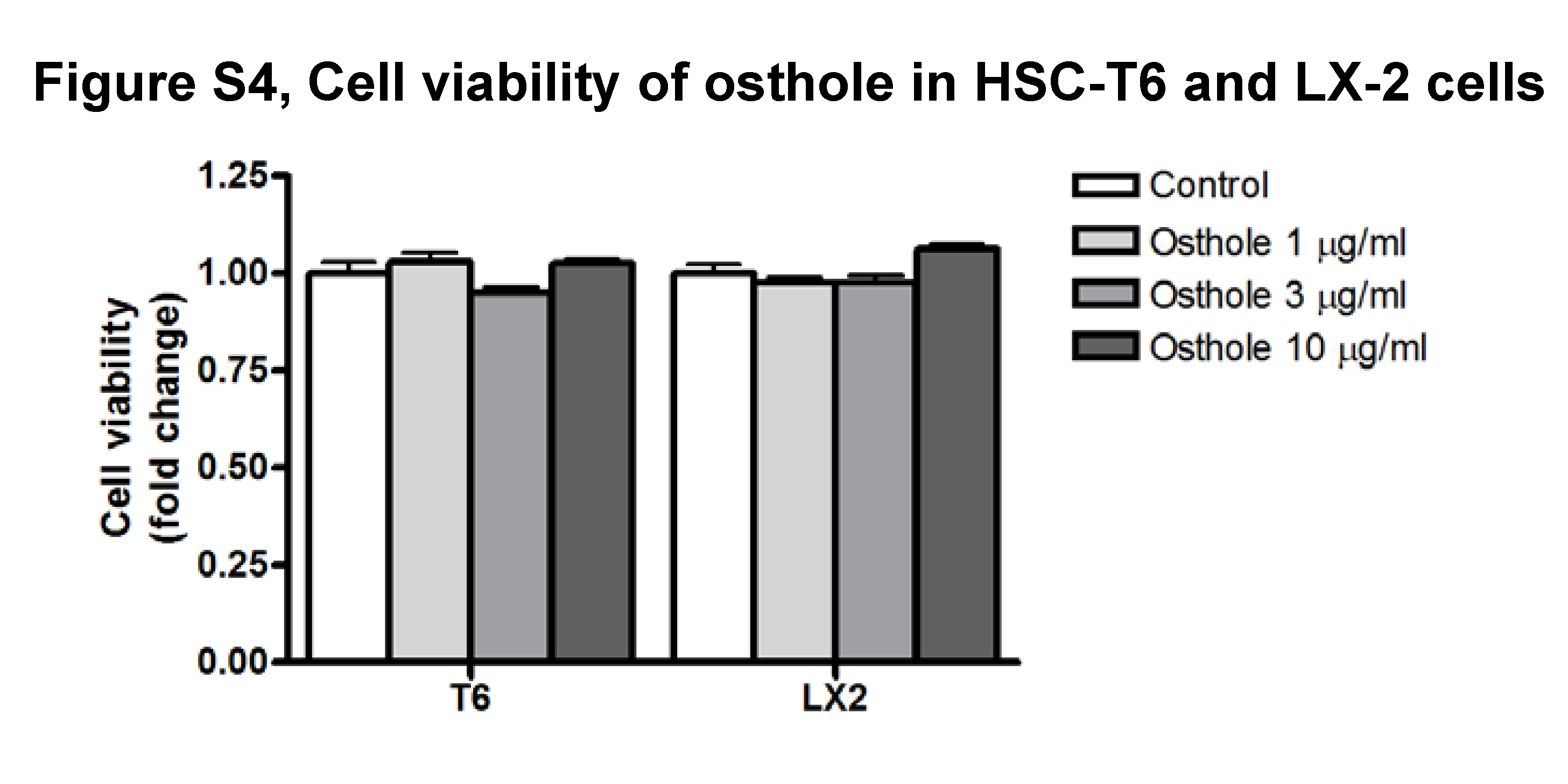

Supplement: Additional file 5: Figure S4. — Cell viability of osthole in HSC-T6 and LX-2 cells. There was no cytotoxicity of osthole (1, 3, and 10 μg/ml) in HSC-T6 and LX-2 cells for 24 h. (TIFF 746 kb) [file 12929_2015_168_MOESM5_ESM.tiff]
